# The Effects of Weather and Climate Change on Dengue

**DOI:** 10.1371/journal.pntd.0002503

**Published:** 2013-11-14

**Authors:** Felipe J. Colón-González, Carlo Fezzi, Iain R. Lake, Paul R. Hunter

**Affiliations:** 1 The Abdus Salam International Centre for Theoretical Physics, Earth System Physics Section, Trieste, Italy; 2 Tyndall Centre for Climate Change Research, School of Environmental Sciences, University of East Anglia, Norwich, United Kingdom; 3 School of Environmental Sciences, University of East Anglia, Norwich, United Kingdom; 4 Department of Economics, University of California, San Diego, La Jolla, California, United States of America; 5 Norwich Medical School, University of East Anglia, Norwich, United Kingdom; U.S. Naval Medical Research Unit No. 2, Indonesia

## Abstract

**Background:**

There is much uncertainty about the future impact of climate change on vector-borne diseases. Such uncertainty reflects the difficulties in modelling the complex interactions between disease, climatic and socioeconomic determinants. We used a comprehensive panel dataset from Mexico covering 23 years of province-specific dengue reports across nine climatic regions to estimate the impact of weather on dengue, accounting for the effects of non-climatic factors.

**Methods and Findings:**

Using a Generalized Additive Model, we estimated statistically significant effects of weather and access to piped water on dengue. The effects of weather were highly nonlinear. Minimum temperature (Tmin) had almost no effect on dengue incidence below 5°C, but Tmin values above 18°C showed a rapidly increasing effect. Maximum temperature above 20°C also showed an increasing effect on dengue incidence with a peak around 32°C, after which the effect declined. There is also an increasing effect of precipitation as it rose to about 550 mm, beyond which such effect declines. Rising access to piped water was related to increasing dengue incidence. We used our model estimations to project the potential impact of climate change on dengue incidence under three emission scenarios by 2030, 2050, and 2080. An increase of up to 40% in dengue incidence by 2080 was estimated under climate change while holding the other driving factors constant.

**Conclusions:**

Our results indicate that weather significantly influences dengue incidence in Mexico and that such relationships are highly nonlinear. These findings highlight the importance of using flexible model specifications when analysing weather–health interactions. Climate change may contribute to an increase in dengue incidence. Rising access to piped water may aggravate dengue incidence if it leads to increased domestic water storage. Climate change may therefore influence the success or failure of future efforts against dengue.

## Author Summary

Relationships between weather and mosquito-borne diseases are nonlinear in nature. This means that the number of disease cases does not vary equally with changes in the climate system. Identifying adequately the form of the relationship between disease outcomes and their drivers in an empirical fashion can be tedious and imprecise. Here, we use a statistical modelling approach that estimates the form of the relationships between dengue and weather in an automated way. We use this approach to analyse a comprehensive dataset covering 23 years of dengue reports from Mexico. Our model incorporates the effects of some non-climatic factors that are key for disease occurrence. We then use our estimations to project the potential impact of climate change on dengue incidence under three different scenarios for three different time periods. The estimated effects of weather on dengue were highly nonlinear. These results highlight the importance of using flexible modelling approaches for the analysis of disease-weather relationships with a nonlinear behaviour. Rising access to water supply was related to increases in dengue incidence. This situation may be related to increased water storage induced by unreliable water supply. Dengue incidence may increase to about 40% by 2080 due to climate change. This increase in dengue incidence may be aggravated by a rising access to piped water if it leads to domestic water storage, although any adaptation measures to rising dengue may also affect the risk. Our results contribute to a better overall understanding of the epidemiology of dengue.

## Introduction

Dengue is the most widely distributed and rapidly spreading mosquito-borne viral disease in the world [1]. This acute febrile disease affects all age groups [2], and is caused by four antigenically distinct but genetically related viruses (serotypes) [3]. Dengue has become endemic in over 100 countries in Africa, the Americas, the Eastern Mediterranean, South-east Asia and the Western Pacific [1]. Approximately 2.5 billion people are at risk from dengue transmission. About 50 million new dengue infections [1] and at least 12,000 deaths, mainly among children, occur worldwide every year [4]. The economic burden of dengue has been estimated at approximately 2.1 billion US dollars per annum in Latin America and the Caribbean alone [5]. In some regions, such as the Americas, the economic losses caused by dengue are similar to those attributed to malaria and tuberculosis [6]. As there are no specific antiviral medicines treating or vaccines preventing dengue, the only way to manage the disease is through the control of vector populations [7].

The global incidence rate of dengue has substantially increased over the last six decades (from about 900 annual cases reported to WHO over 1955—1959 to about 926 thousand annual cases over 2000—2007) [1], [4] influenced by numerous mechanisms including population growth, unplanned urbanisation, increased travel and transportation of goods, lack of political will and limited resources for implementing effective control measures [7]. The spatial distribution of the main dengue vector, *Aedes aegypti*, has also increased over the last 25 years [8]. Increases in both dengue incidence and *A. aegypti* distribution have also been associated to variations in the climate system, including climate change (see references [9], [10] for an example). The evidence of the effects of climate drivers on dengue incidence is still under debate [8], [11].

This paper estimates the relative effects of weather (minimum and maximum temperature, and precipitation) on dengue accounting for a range of non-climatic factors (e.g. access to piped water, urbanisation, gross domestic product, and long-term trends and seasonality; see Methods). Our model parameters are then used to project the potential effects of climate change on dengue incidence by 2030, 2050 and 2080 under three emission scenarios (A1B, A2, and B1) described by Nakicenovic and Swart [12].

Several empirical models have been developed for estimating the effects of weather on dengue (for example, references [10], [13]), and some of these have been used as a baseline to estimate the potential impacts of climate change on the future distribution and risk of dengue infection (for example, references [10], [14]). However, the majority of these studies have been conducted in small geographical areas, covered relatively short periods of time, and include very limited non-climatic confounders (for example, references [10], [13], [15]) leading to several limitations. For example, small populations commonly result in low disease numbers leading to unstable risk estimations [16]; small areas are also more likely to be climatically and socioeconomically homogeneous [17], [18], making it harder to extrapolate the results to areas with greater climatic or socioeconomic variability.

Our case-study has various unique features that minimize the identified problems. First, we used a large panel of province-specific data with a refined temporal resolution (monthly) covering the entirety of Mexico to investigate a greater geographical area (∼2 million km^2^), time frame (276 months), and number of reported cases (417,668) than previous studies. Second, the great socioeconomic heterogeneity [19] and climatic diversity of Mexico, which includes both tropical and subtropical areas [20], allowed us to estimate robust and generalized relations between dengue, climatic and socioeconomic factors, which may be extrapolated to a large number of regions with similar climatic and socioeconomic features. Third, we control for the effect of potential un-observed confounders (e.g. social behaviour) by incorporating province-specific fixed-effects into our model. Fourth, we allowed for nonlinear relationships between dengue and weather by adopting a semi-parametric modelling approach. Specifically, we implemented a Generalized Additive Model (GAM) coupled with penalized likelihood function and an automated smoothing selection criterion, which estimated the optimal degree of nonlinearity of the model directly from the data [21]. The advantage of this approach is that it determines the model flexibility within the actual estimation process. This method has been described in detail elsewhere [21].

## Methods

### Dengue data

**Figure 1:**
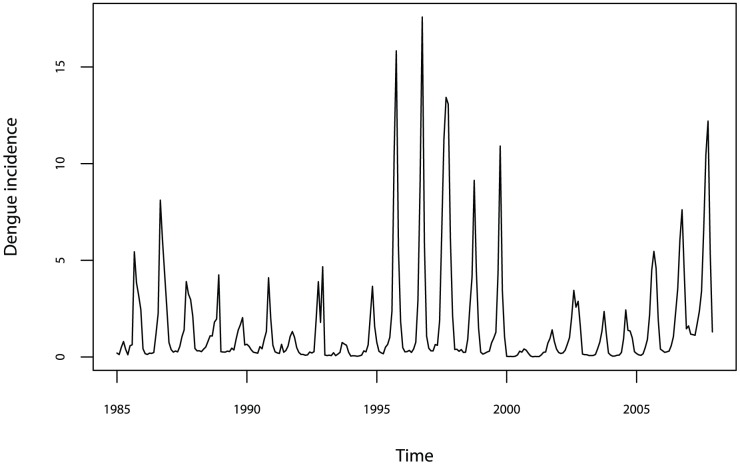
Time series of average monthly dengue incidence in Mexico (cases/100,000 people) over the period January 1985 to December 2007.

Province-specific monthly reports of laboratory confirmed dengue cases were collected from the Mexican National System of Epidemiologic Surveillance [22] for the period 1985—2007 (Figure 1). Dengue and severe dengue cases were aggregated because they correspond to different presentations of the disease.

### Weather data

We obtained the province-specific monthly mean values of minimum temperature, average maximum temperature and monthly accumulated precipitation from the Mexican National Meteorological Service for each province for the period 1971—2007. These monthly mean values were computed using province-specific observations from all available meteorological stations across Mexico. The network of meteorological stations from the National Meteorological Service consists of over 2,000 stations distributed across the whole country. The frequency and duration of rainy events were not considered as such information was not available from the National Meteorological Service. We preferred station data over satellite data because meteorological stations seem to provide more reliable information about the conditions (particularly rainfall) of an area than do satellites [23], [24].

Because the modulating effects of the climate system on vector populations do not immediately result in changes on dengue transmission, we specified meteorological variables within biologically and physically plausible time lags based on literature reports in Mexico (for example, references [25], [26], [27]), and considering the delays in laboratory confirmation of suspected cases and their reporting. Optimal lags for the climatic variables comprised monthly average minimum temperature, average monthly maximum temperature and accumulated monthly precipitation lagged one and two months. Due to significant autocorrelation between the lags of these variables (Spearman's rank rho 0.7), we created new variables (Tmin_1∶2_, Tmax_1∶2_, and Precipitation_1∶2_) taking the mean of the values of the two optimal lags.

### Socioeconomic data

Provincial population data were retrieved from the National Institute of Statistics and Geography (INEGI) [28] for 1990, 1995, 2000 and 2005. The proportion of the population with access to piped water was also obtained from INEGI for 1990, 2000, 2005 and 2010. The share of the population living in urban areas (urbanisation) was obtained from the Chamber of Deputies [29] for 1980, 1990, 1995, 2000 and 2004. Intervening years for these variables were estimated using linear interpolation. Yearly GDP per capita was obtained from the World Bank for the whole period of study. These data were then deflated at constant 2003 values. GDP data were originally aggregated at the national level. To obtain province-specific GDP estimates we assumed that the proportion of the national GDP for each province was the same as that for which GDP information were available (1993–2005) from INEGI. Thus, the province-specific proportion of GDP for the period 1985–1993 was assumed to be the same than that observed in 1993 on the INEGI data. Proportions for the subsequent years were assumed to be the same as in the INEGI data.

### Climate change data

To project the potential impact of climate change on dengue (with Monte Carlo 95% confidence intervals), we retrieved province-specific historical values (relative to the 1970—1999 climatology) and projected changes for the years 2030, 2050 and 2080 under three climate change scenarios (A1B, A2 and B1) for monthly mean temperature and precipitation from the National Institute of Ecology [30] using the coordinates of the centroids of each province. The coordinates of the centroids were obtained using the standard ‘coordinates’ routine within the ‘sp’ package for R [31] and a digital map of Mexico. Average monthly minimum temperature, maximum temperature and precipitation were estimated as the monthly averages of the baseline period based on the observational data obtained from the Mexican National Meteorological Service. To generate new temperature values for each scenario, we added the corresponding projected changes to the historical values. Precipitation was rescaled multiplying the historical value by the corresponding projected percentage of variation. Average minimum and maximum temperature, and accumulated precipitation values (historical and projected) lagged 1 and 2 months were then used for the climate change projections.

### GAM analysis

We specified the expected number of dengue cases during month *t* and province *i* as:
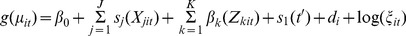
(1)where *g*(.) is a log link function of the expectation *μ*_it_≡*E*(*Y_it_*), with *Y_it_* as the series of dengue counts. *X_jit_* denotes the *j*-th meteorological variables, *s_j_*(.) and *s_1_*(.) are smooth functions for the meteorological variables and the time trend defined via penalized cubic regressions splines; *Z_kit_* denotes the *k*-th socio-economic variables (GDP per capita, proportion of people living in urban areas, proportion of people with access to piped water) which enter the model linearly; *d_i_* are province-specific fixed effects [32] to capture the effects of potential unobserved confounders (e.g. social behaviour) in the model. Log(*ξ_it_*) indicates the logarithm of the population/month at risk included as an offset variable. This offset variable standardises dengue occurrence by population to compute estimations on the incidence rate rather than on the total number of dengue cases. To account for possible over-dispersion, we allowed the scale parameter to be different from the mean [33]. This led to a quasi-maximum likelihood Poisson model, which is the standard consistent estimator for count variables [34].

The province-specific fixed effects control for province-specific omitted variable bias and un-modelled confounders such as social behaviour. The smooth function of time controls for long-term trends and seasonality that could arise from non-climatic factors such as resistance of the vector to insecticides, changes in the diagnostic techniques, holidays and seasonal water storage practices. To ensure the robustness of our results, we tested other specifications to account for long-term and seasonal trends including: 0,1 categorical variables for each year and for each season for the period, categorical variables for each year with a sinusoidal term for seasonal trends, and a linear trend with a sinusoidal term for seasonal trends. As is the case of the smooth function for time, our categorical variables account for long-term and seasonal changes that could happen as a consequence of confounding non-climatic factors such as seasonal water storage or year-specific disruptions in the public health systems. Estimations were conducted using the ‘mgcv’ package [21] for R version 2.12.0 [31].

The smooth functions are represented by regression splines, which can be written as linear-combinations of known basis functions of the regressors.
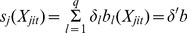
(2)where *b_l_*(.) denotes the basis functions and *δ_l_* the parameters to be estimated. The number of basis functions *q* determines the maximum possible flexibility of the relation between *X_jit_* and *g*(*μ_it_*) (see Equation 1); the greater the value of *q*, the more flexible is the estimated effect. Here, we used Cubic Regression Splines (CRS) in which the basis functions *b_l_*(.) are constructed by dividing the range of values of the independent variable into segments separated by knots. A local cubic regression is fitted to each segment. The continuity and smoothness at the knots is ensured imposing conditions on the first and second-order derivatives [35]. Our estimation method, implemented via Penalized Iteratively Reweighted Least Squares (P-IRLS) is designed to automatically reduce the nonlinearities not supported by the data to simple linear forms [21].

### Climate change scenarios

We generated extrapolations of projected dengue risk based on our fitted GAM parameters for the years 2030, 2050 and 2080, under the A1B, A2 and B1 climate change scenarios. The storylines behind these scenarios are described in detail elsewhere [12]. Briefly, the A1B scenario relates to a future with very rapid economic growth, global population peaking in mid-century, and the introduction of more efficient technologies with a balance in energy-sources-related technological change [12]. The A2 scenario describes a future with a continuously increasing global population, economic development regionally oriented, and a slower and fragmented per capita economic growth and technological change than other scenarios [12]. Lastly, the B1 scenario considers a similar global population growth as the A1B, but with an economic structure towards a service and information economy, reductions in material intensity, and the introduction of clean and resource-efficient technologies [12].

We retrieved province-specific temperature and precipitation ensemble outputs of multiple models (relative to the base period climatology of 1970–1999) from the website of the Mexican National Institute of Ecology for the years 2030, 2050 and 2080. The methodology and outputs of these ensembles have been described by Magaña and Caetano [36]. Briefly, the three scenarios (A1B, A2, and B1) describe rising temperature at an increasing rate all over the country. The north-west region is the most greatly affected by temperature at the end of the century [36]. In these scenarios, changes in precipitation are be very irregular; though they agree that decreases are expected mainly in the north and north-west, followed by the Yucatán Peninsula and central Mexico [36]. Changes in both temperature and precipitation are expected to be greater under the A2 scenario (high emissions) followed by the A1B and B1 [36].

To conduct our estimations, we used future projections of climate holding all the other driving forces constant (fixed to the baseline year 2000) to isolate the effects of climate on dengue. While our model is robust to the confounding effects of observed and un-observed non-climatic factors, these projections are not predictions of the future but rather aim to show the potential impact of climate change on dengue incidence whilst keeping the other driving forces constant.

## Results

### GAM analysis

Table 1 presents the estimates of our Poisson GAM for dengue incidence per province. This specification explained 61% of the deviance of dengue incidence. The high values of the effective degrees of freedom (*edf*) of the smooth functions indicate that associations between dengue and weather are highly nonlinear. The effects of all meteorological variables and access to piped water on dengue were found to be significant. The estimate of the scale parameter was very high (>80) indicating over-dispersed data. The results presented here were robust to other model specifications with different controls for long-term and seasonal trends (Table S1).

**Table 1:** Model estimates of the effects of weather and socioeconomic development on dengue across Mexico.

Smooth terms	*edf* (F )
*s*(Time)	**44.187 (49.880)**
*s*(Tmin_1∶2_)	**3.820 (27.530)**
*s*(Tmax_1∶2_)	**2.958 (52.870)**
*s*(Precipitation_1∶2_)	**3.570 (76.580)**

Linear terms	Estimate (SE)
Constant	**−23.270 (2.975)**
Access to piped water	**0.052 (0.007)**
Urbanisation	0.016 (0.010)
GDP per capita	−1.393 (0.869)

Model statistics	
**Fixed-effects**	**Included**
Log-Likelihood	−398002.5
Explained deviance	61%
GCV	88.727
Scale parameter	85.459

Values in bold font were significant at the 0.001 level. *edf* = effective degrees of freedom of the smooth function terms (*edf*>1 indicate nonlinear relationships); F-value is an approximate F-test as in [21], maximum number of spline basis for the meteorological terms = 5. SE = approximate asymptotic standard error; GCV = Generalized Cross Validation. Estimation was performed via Penalized Iteratively Reweighted Least Squares (P-IRLS) and GCV score minimization by outer iteration.

**Figure 2:**
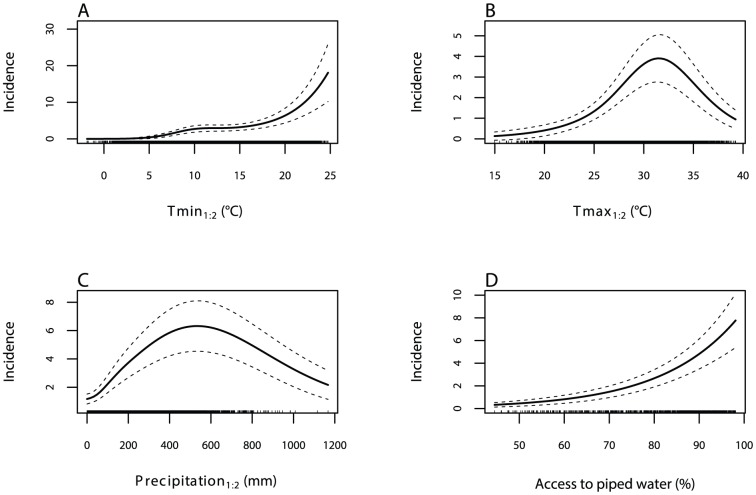
GAM-estimated relationships. The figure shows the GAM estimated relationships between average monthly dengue incidence and (A) Tmin_1∶2_, (B) Tmax_1∶2_, (C) Precipitation_1∶2_, and (D) the proportion of the population with access to piped water. Solid lines indicate the average expected number of dengue cases (cases/100,000 people per month); dashed lines indicate the estimated 95% Bayesian estimation confidence intervals.

Figure 2 depicts the relationships estimated by our model. Figure 2A shows almost zero response of dengue to Tmin_1∶2_ below 5°C, a modest increased response above this threshold, and then a rapid increasing response when temperatures rise above 18°C. Dengue incidence also increases gradually with rising Tmax_1∶2_ (Fig. 2B), showing a peak at approximately 32°C after which the response declines. Figure 2C shows a quadratic relationship between dengue incidence and Precipitation_1∶2_ with a plateau at approximately 550—650 mm. Figure 2D depicts a positive relationship between dengue incidence and the proportion of the population with access to piped water, indicating that as access to piped water rises so too does dengue. Urbanisation and GDP did not show a significant association with dengue. This may indicate that these variables do not play a key role in determining dengue transmission in Mexico or that our data, after the removal of time invariant characteristics by the province-specific fixed-effects, do not contain enough variability for estimating meaningful relationships for these variables.

Higher incidence rates were observed during the wet season (May to October) compared to the rest of the year. We compared the model estimates with the observed data for the whole year, the wet and dry seasons. The model captured much of the spatiotemporal variability observed in dengue incidence (see Figure S1) providing evidence that our estimates are robust. We tested the influence of the province with the greatest incidence rate on the model fit excluding it from the model. The results presented in this paper were robust to these changes.

### Climate change projections

Our projections suggest that mean annual dengue incidence may increase by about 12—18% by 2030, 22—31% by 2050, and 33—42% by 2080 across Mexico showing an increasing effect of climate change on dengue (Table 2). Such positive and increasing impact of climate change on dengue cases is also evident at the province level. As an illustration, we report the results obtained for the Mexican provinces of Nuevo León, Querétaro and Veracruz. These provinces not only have very different climatic regimes, but also show different levels of endemicity. Veracruz is an endemic province with very regular seasonal transmission and a warm and humid climate. Nuevo León is endemic but with periods of very low or no transmission during the dry season, and it has a semi-warm semi-dry climate. Querétaro, on the other hand, is epidemic-prone with very intermittent transmission, and a temperate semi-dry climate. Further information on the province-specific projections for the whole country can be found on Table S2. Our projections indicate that in already endemic provinces (Nuevo León and Veracruz) we observe a very significant raise in dengue cases, (from 1.7 to about 2.4 reported cases/100,000 people in Nuevo Leon; and from 2.6 to about 4.2 reported cases/100,000 people in Veracruz annually). On the other hand, in epidemic-prone provinces (Querétaro), we do not observe a significant increase in transmission; with dengue cases remaining uncommon (from 0.04 to about 0.08 reported cases/100,000 people annually).

**Table 2:** GAM-estimated average annual dengue incidence under climate change.

Region	Baseline (95% CI)	Scenario	2030 (95% CI)	2050 (95% CI)	2080 (95% CI)
National	1.001 (0.708–1.466)	A1B	1.177 (0.832–1.723)	1.315 (0.926–1.961)	1.411 (1.001–2.078)
National	1.001 (0.708–1.466)	A2	1.118 (0.798–1.640)	1.258 (0.894–1.863)	1.412 (1.016–2.093)
National	1.001 (0.708–1.466)	B1	1.149 (0.814–1.702)	1.222 (0.870–1.813)	1.333 (0.942–2.003)
Nuevo León	1.683 (1.141–2.589)	A1B	2.092 (1.427–3.208)	2.296 (1.555–3.510)	2.539 (1.757–3.874)
Querétaro	0.042 (0.014–0.138)	A1B	0.056 (0.018–0.179)	0.067 (0.022–0.227)	0.082 (0.026–0.278)
Veracruz	2.630 (1.801–3.961)	A1B	3.358 (2.292–5.067)	3.388 (2.653–5.901)	4.470 (3.104–6.836)
Nuevo León	1.683 (1.141–2.589)	A2	2.001 (1.360–3.082)	2.240 (1.520–3.430)	2.654 (1.801–4.043)
Querétaro	0.042 (0.014–0.138)	A2	0.053 (0.017–0.181)	0.067 (0.020–0.223)	0.085 (0.026–0.274)
Veracruz	2.630 (1.801–3.961)	A2	3.005 (2.067–4.568)	3.377 (2.556–5.731)	4.289 (2.934–6.578)
Nuevo León	1.683 (1.141–2.589)	B1	1.950 (1.330–2.997)	2.248 (1.539–3.399)	2.392 (1.617–3.601)
Querétaro	0.042 (0.014–0.138)	B1	0.056 (0.018–0.200)	0.062 (0.194–0.202)	0.072 (0.023–0.251)
Veracruz	2.630 (1.801–3.961)	B1	3.250 (2.235–4.892)	3.522 (2.399–5.363)	4.216 (2.823–6.345)

The values for each climate change scenario represent the difference in mean annual dengue incidence (expressed in cases/100,000 people) relative to the 1970–1999 baseline scenario. Confidence intervals were generated with 5,000 Monte Carlo repetitions.

**Figure 3:**
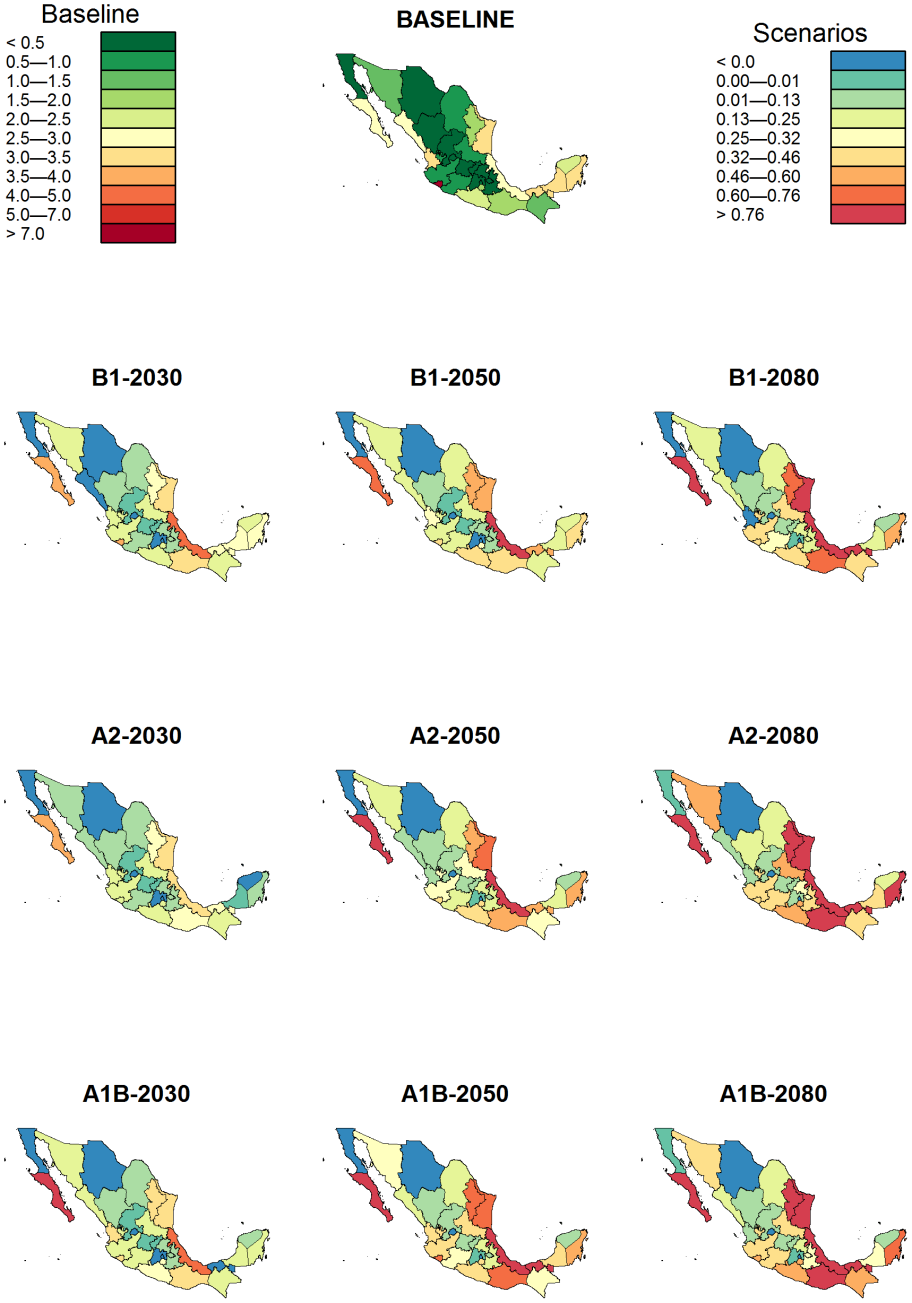
Changes in annual dengue incidence under climate change. The figure shows the GAM-estimated 1970–1999 average annual dengue incidence (cases/100,000 people) across Mexico for the baseline scenario (top), and the estimated difference in mean annual dengue incidence relative to that baseline (cases/100,000 people) by 2030, 2050, and 2080 under the A1B, A2, and B1 climate change scenarios.

Figure 3 shows that the majority of provinces across Mexico are expected to undergo an increase in dengue transmission under future climate change. The difference in mean annual dengue incidence between the projections and the baseline scenario are likely to be greater in endemic provinces (with year-round transmission or with periods of no transmission during the dry season), and particularly stronger in southern and eastern provinces characterized by warm and humid climates. However, our projections show significant spatial heterogeneity with some north-western provinces and the north of the Yucatán Peninsula being likely to observe decreases in dengue incidence by 2080 presumably due to the impact of reduced precipitation on the creation of breeding sites [8], [37], [38].

## Discussion

In this study we have presented an analysis of the association between dengue incidence and climatic variables in Mexico. We then used the model generated to make projections about the impact of future climate change on dengue incidence. For this work we brought together one of the longest (276 months of dengue reports) and more spatially diverse (nine major climatic regions across ∼2 million km^2^) dengue and climate datasets yet assembled. We furthermore used an analytic approach (Generalized Additive Modelling) that is specifically designed to analyse data when the impact of the predictors on the outcome variables are nonlinear. GAMs coupled with penalized estimation provide a more flexible modelling approach than conventional regression methods, allowing the specification of flexible functional forms with the degree of non-linearity estimated directly from the data [21]. This characteristic of GAMs resolves the subtle task of determining the model flexibility a priori [21].

We showed that weather significantly influences dengue incidence in Mexico. However, all these relationships are highly nonlinear. Tmin_1∶2_ has the biggest impact on dengue with almost zero risk below 5°C, a modest increased risk above this temperature, and a rapid increasing risk when average minimum temperatures rise above 18°C. The sharp increase in dengue incidence at minimum temperatures beyond 18°C provides a partial explanation for the strong seasonality observed in tropical provinces where seasonal variations in temperature are not greater than a few degrees [39]. These effects are consistent with the biology of both the dengue vector and the dengue virus because rising temperatures shorten the extrinsic incubation period (EIP) of the virus, as well as the development time and the gonotrophic cycle of the mosquito resulting in an increased likelihood of dengue transmission [8], [39], [40].

Maximum temperature also has an effect independently from Tmin. The risk of dengue increases as Tmax_1∶2_ rises above about 20°C to a peak around 32°C after which the risk declines. The decay in the response of dengue to high levels of Tmax_1∶2_ may be explained by the maximum transmission efficiency of *A. aegypti* achieved above 32°C [40], and by adult mosquitoes gradually dying at temperatures above 36°C [39].

There is also an increasing risk as Precipitation_1∶2_ rises to about 550 mm beyond which risk declines. The progressive increase in dengue incidence at low Precipitation_1∶2_ levels suggests the creation of rain-filled (outdoors) breeding sites, whereas the decay observed at high levels, may be due to the wash-out of such breeding sites [37].

Our findings regarding the impact of weather on dengue risk are consistent with the results of other studies using empirical modelling (for example, [10], [41], [42], [43]). However, previous studies using OLS, GLM, or ARIMA methods are unlikely to have fully captured the nonlinearities that we have demonstrated. Also, because of the larger database over more climate zones, we have been able to model these relationships over the whole range of climate variations likely to be seen under future climate change. Our results should therefore be generalizable to other regions and climatic zones and provide a better basis for modelling the impact of future climate change.

Also of interest is the significant association between dengue incidence and the proportion of the population with access to piped water. This finding is at odds with previous observations suggesting that piped water supply was protective [15]. Schmidt and colleagues suggested that dengue risk was higher in people without access to piped water supply because of the need to store water, and mosquitoes could then breed in this stored water. However, paradoxically they showed that people using rainwater harvesting had the lowest adjusted risk, and one would expect these people to store most water. It may be that in Mexico people reliant on piped water have intermittent water delivery making water storage necessary, hence providing mosquito breeding sites. The relationship between water supply and dengue risk is not simple and may differ from one locality to another.

We project an increase of up to ∼40% in dengue incidence in Mexico by 2080 due to climate change, holding the other driving factors constant. These estimations were computed considering the projected changes in monthly mean temperature and precipitation. Downscaled projections on the intensity and variability of rainy events in Mexico are, to our knowledge, currently unavailable at this point in time.

Based on the number of cases reported each year in the dataset, this would equate to about 7,000 extra cases reported each year. However, actual excess disease burden will be greater than this value would suggest. Previous research has shown that for every official dengue report included in surveillance, there are 10—27 cases unreported [44], [45]. Consequently, the real increase may be of the order of 70,000 to 189,000 extra cases per year. In addition, there may be many more asymptomatic infections. There is an even bigger concern in that this increase in infections, both symptomatic and asymptomatic may increase the incidence of the more severe forms of dengue. Previous research in the field has demonstrated that secondary infection with a new serotype increases the risk of severe dengue (see for example [3], [46], [47]). Therefore, if dengue incidence increases under climate change, the severe-dengue∶dengue ratio may potentially increase.

Although our projections suggest that dengue incidence may increase in the long run, they have been computed to show the potential impact of climate change on dengue incidence whilst holding the other driving forces constant. Therefore, the projected increasing trends in dengue incidence may be different in the presence of adaptation strategies (e.g. changes in water storage technologies, as well as water supply practices and systems) to alleviate the adverse effects of climate change. The assessment of this hypothesis is beyond the scope of this study.

In conclusion, we have reported on the association between dengue incidence in Mexico and climate variables using one of the longest and more spatially diverse dengue and climate datasets yet assembled. We argue that our results provide a much improved empirical model of the relationship between the dengue and climate than has been presented to date, because of the much longer data set and the use of GAM regression to better model the nonlinear nature of the relationships. Such an improved model is critical to help make better estimations of the impact of climate change on dengue into the future. Consequently, we further argue that this dataset can be used to draw conclusions about the relationship between dengue and weather regions with similar climatic and socioeconomic features. We have estimated the impact that future climate change will increase dengue incidence by about 40%, but that the proportional increase in severe dengue forms may be greater.

## Supporting Information

Figure S1 Observed vs. GAM-estimated mean monthly dengue incidence. The figure shows a comparison between the observed and GAM-estimated mean monthly dengue incidence across Mexico during the whole year (upper), wet season (middle), and dry season (lower). The wet season occurs between November–April, and the dry season between May–Oct. Incidence is expressed in cases/100,000 people.
(TIFF)

Table S1 Model estimates using different representations of long-term and seasonal trends. Values in bold font were significant at the 0.001 level. The original model is as in Equation 1. Model 2 replaces the smooth variable of time (Equation 1) with categorical variables for calendar year and month. Model 3 uses categorical variables for calendar year and season. Model 4 includes a categorical variable for calendar year and a sinusoidal term for season. The sinusoidal term can be expressed as *sin*(2×π×time/12)+*cos*(2×π×time/12), where time is an index variable 1,…,*n*. Model 5 includes a linear trend and a sinusoidal function identical to that for Model 4.
(DOC)

Table S2 Province-specific GAM-estimated average annual dengue incidence (per 100,000 people) under climate change. The national average annual values are included as a reference. Confidence intervals were generated with 5,000 Monte Carlo repetitions.
(DOC)
