# Supplementary figures and images for: The Effects of Weather and Climate Change on Dengue
Source: PLoS Negl Trop Dis. 2013 Nov 14;7(11):e2503. doi: 10.1371/journal.pntd.0002503 (PMC3828158; doi:10.1371/journal.pntd.0002503)

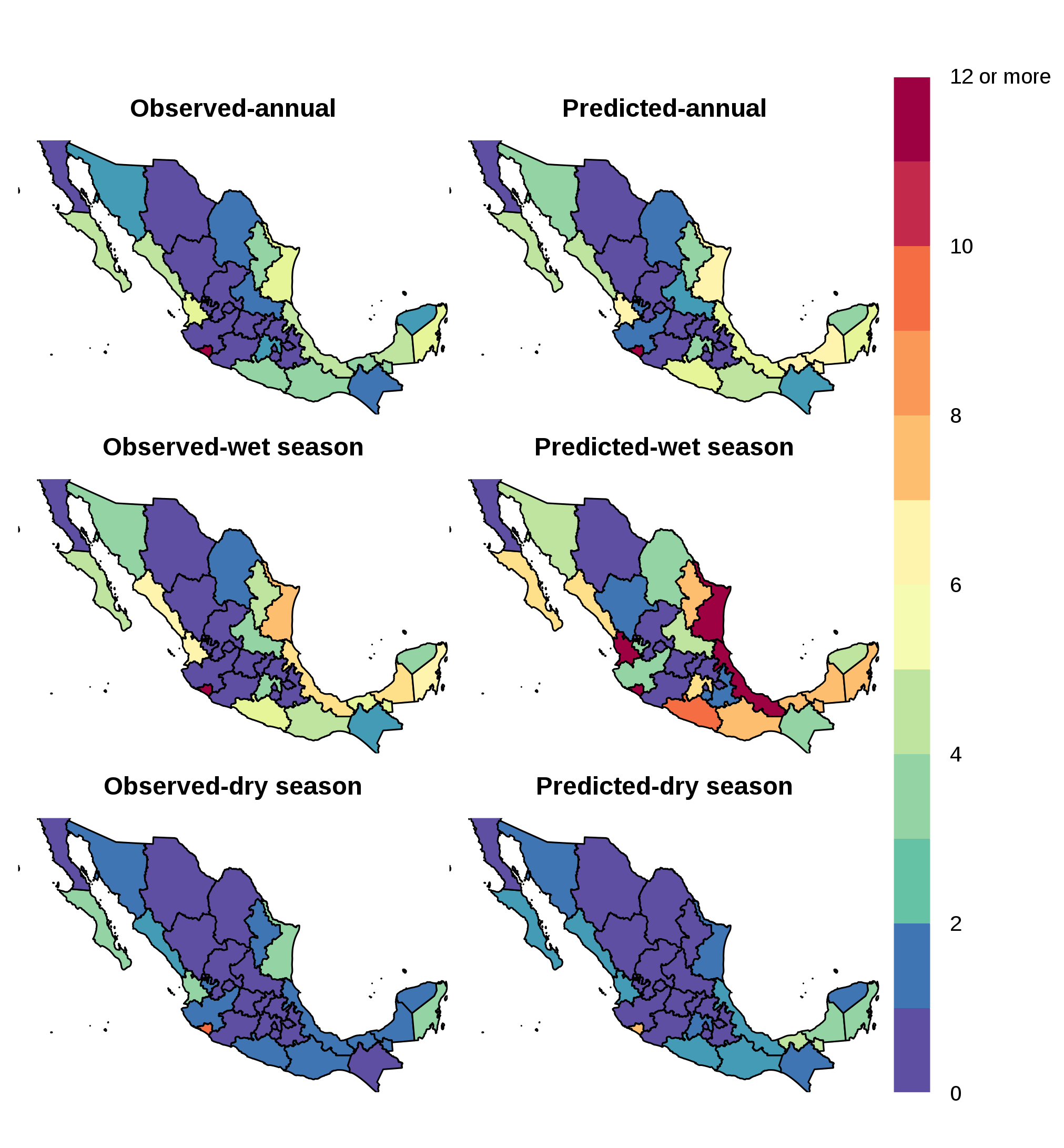

Supplement: Figure S1 — Observed vs. GAM-estimated mean monthly dengue incidence. The figure shows a comparison between the observed and GAM-estimated mean monthly dengue incidence across Mexico during the whole year (upper), wet season (middle), and dry season (lower). The wet season occurs between November–April, and the dry season between May–Oct. Incidence is expressed in cases/100,000 people. (TIFF) [file pntd.0002503.s001.tif]
