# Supplementary material for: The Effects of Weather and Climate Change on Dengue
Source: PLoS Negl Trop Dis. 2013 Nov 14;7(11):e2503. doi: 10.1371/journal.pntd.0002503 (PMC3828158; doi:10.1371/journal.pntd.0002503)
Supplement: Table S1 — Model estimates using different representations of long-term and seasonal trends. Values in bold font were significant at the 0.001 level. The original model is as in Equation 1. Model 2 replaces the smooth variable of time (Equation 1) with categorical variables for calendar year and month. Model 3 uses categorical variables for calendar year and season. Model 4 includes a categorical variable for calendar year and a sinusoidal term for season. The sinusoidal term can be expressed as sin(2×π×time/12)+cos(2×π×time/12), where time is an index variable 1,…,n. Model 5 includes a linear trend and a sinusoidal function identical to that for Model 4. (DOC) [file pntd.0002503.s002.doc]

| **Specification** | **Original model** | **Model 2** | **Model 3** | **Model 4** |
| --- | --- | --- | --- | --- |
| Smoothers (*edf*) | | | | |
| *s*(Tmin_1:2_) | **3.820** | **3.561** | **3.367** | **3.802** |
| *s*(Tmax_1:2_) | **2.958** | **2.958** | **3.021** | **2.972** |
| *s*(Precipitation_1:2_) | **3.570** | **3.709** | **3.793** | **3.448** |
| Linear terms (coefficients) | | | | |
| Access to piped water | **0.052** | **0.063** | **0.021** | **0.063** |
| Urbanisation | 0.016 | 0.005 | **0.039** | 0.017 |
| GDP per capita | -1.393 | -2.857 | 0.026 | -3.365 |
| Model statistics | | | | |
| Explained deviance | 61% | 59% | 63% | 53% |
| GCV | 88.727 | 95.212 | 86.056 | 108.488 |
