# Supplementary material for: The Effects of Weather and Climate Change on Dengue
Source: PLoS Negl Trop Dis. 2013 Nov 14;7(11):e2503. doi: 10.1371/journal.pntd.0002503 (PMC3828158; doi:10.1371/journal.pntd.0002503)
Supplement: Table S2 — Province-specific GAM-estimated average annual dengue incidence (per 100,000 people) under climate change. The national average annual values are included as a reference. Confidence intervals were generated with 5,000 Monte Carlo repetitions. (DOC) [file pntd.0002503.s003.doc]

| **Region** | **Baseline (95% CI)** | **Scenario** | **2030 (95% CI)** | **2050 (95% CI)** | **2080 (95% CI)** |
| --- | --- | --- | --- | --- | --- |
| National | 1.001 (0.708-1.466) | A1B | 1.177 (0.832-1.723) | 1.315 (0.926-1.961) | 1.411 (1.001-2.078) |
| National | 1.001 (0.708-1.466) | A2 | 1.118 (0.798-1.640) | 1.258 (0.894-1.863) | 1.412 (1.016-2.093) |
| National | 1.001 (0.708-1.466) | B1 | 1.149 (0.814-1.702) | 1.222 (0.870-1.813) | 1.333 (0.942-2.003) |
| Aguascalientes | 0.002 (0.000-0.239) | A1B | 0.003 (0.000-0.338) | 0.004 (0.000-0.041) | 0.005 (0.000-0.054) |
| Aguascalientes | 0.002 (0.000-0.239) | A2 | 0.003 (0.000-0.304) | 0.004 (0.000-0.398) | 0.005 (0.000-0.535) |
| Aguascalientes | 0.002 (0.000-0.239) | B1 | 0.003 (0.000-0.331) | 0.003 (0.000-0.342) | 0.004 (0.000-0.460) |
| Baja California | 0.008 (0.001-0.050) | A1B | 0.011 (0.002-0.064) | 0.013 (0.002-0.077) | 0.016 (0.003-0.099) |
| Baja California | 0.008 (0.001-0.050) | A2 | 0.010 (0.002-0.061) | 0.012 (0.002-0.071) | 0.015 (0.003-0.090) |
| Baja California | 0.008 (0.001-0.050) | B1 | 0.010 (0.002-0.060) | 0.011 (0.002-0.073) | 0.012 (0.002-0.075) |
| Baja California Sur | 2.845 (1.907-4.445) | A1B | 3.615 (2.443-5.549) | 3.878 (2.634-5.993) | 4.337 (2.937-6.636) |
| Baja California Sur | 2.845 (1.907-4.445) | A2 | 3.337 (2.248-5.158) | 3.658 (2.480-5.671) | 4.276 (2.903-6.581) |
| Baja California Sur | 2.845 (1.907-4.445) | B1 | 3.345 (2.245-5.187) | 3.525 (2.378-5.396) | 3.811 (2.585-5.860) |
| Campeche | 3.046 (2.098-4.561) | A1B | 3.185 (2.193-4.781) | 3.429 (2.372-5.150) | 3.350 (2.327-5.072) |
| Campeche | 3.046 (2.098-4.561) | A2 | 3.053 (2.106-4.566) | 3.254 (2.253-4.860) | 3.412 (2.381-5.208) |
| Campeche | 3.046 (2.098-4.561) | B1 | 3.320 (2.290-5.046) | 3.295 (2.295-4.903) | 3.264 (2.254-4.917) |
| Chiapas | 1.003 (0.694-1.507) | A1B | 1.188 (0.818-1.788) | 1.317 (0.901-2.014) | 1.478 (1.011-2.268) |
| Chiapas | 1.003 (0.694-1.507) | A2 | 1.175 (0.810-1.770) | 1.260 (0.862-1.925) | 1.401 (0.948-2.115) |
| Chiapas | 1.003 (0.694-1.507) | B1 | 1.169 (0.805-1.794) | 1.240 (0.852-1.874) | 1.331 (0.917-2.020) |
| Chihuahua | 0.009 (0.003-0.031) | A1B | 0.012 (0.004-0.038) | 0.013 (0.004-0.042) | 0.014 (0.005-0.044) |
| Chihuahua | 0.009 (0.003-0.031) | A2 | 0.011 (0.003-0.036) | 0.012 (0.004-0.041) | 0.014 (0.005-0.049) |
| Chihuahua | 0.009 (0.003-0.031) | B1 | 0.011 (0.004-0.039) | 0.012 (0.004-0.038) | 0.013 (0.004-0.042) |
| Coahuila | 0.508 (0.331-0.813) | A1B | 0.615 (0.402-0.978) | 0.674 (0.446-1.069) | 0.754 (0.494-1.177) |
| Coahuila | 0.508 (0.331-0.813 | A2 | 0.600 (0.392-0.955) | 0.673 (0.446-1.067) | 0.756 (0.502-1.203) |
| Coahuila | 0.508 (0.331-0.813 | B1 | 0.585 (0.383-0.942) | 0.650 (0.429-1.022) | 0.706 (0.468-1.113) |
| Colima | 7.374 (5.249-10.752) | A1B | 7.810 (5.545-11.441) | 8.007 (5.644-11.945) | 7.949 (5.623-11.802) |
| Colima | 7.374 (5.249-10.752) | A2 | 7.616 (5.415-11.127) | 7.785 (5.489-11.550) | 7.744 (5.411-11.603) |
| Colima | 7.374 (5.249-10.752) | B1 | 7.868 (5.568-11.628) | 7.783 (5.484-11.332) | 7.762 (5.457-11.409) |
| Distrito Federal | 0.001 (0.000-0.012) | A1B | 0.001 (0.000-0.002) | 0.001 (0.000-0.023) | 0.001 (0.000-0.031) |
| Distrito Federal | 0.001 (0.000-0.012) | A2 | 0.001 (0.000-0.017) | 0.001 (0.000-0.021) | 0.002 (0.000-0.035) |
| Distrito Federal | 0.001 (0.000-0.012) | B1 | 0.001 (0.000-0.017) | 0.001 (0.000-0.022) | 0.001 (0.000-0.026) |
| Durango | 0.135 (0.075-0.252) | A1B | 0.184 (0.103-0.342) | 0.217 (0.122-0.404) | 0.261 (0.147-0.485) |
| Durango | 0.135 (0.075-0.252) | A2 | 0.170 (0.095-0.317) | 0.200 (0.112-0.373) | 0.284 (0.163-0.527) |
| Durango | 0.135 (0.075-0.252) | B1 | 0.166 (0.093-0.314) | 0.182 (0.103-0.338) | 0.216 (0.122-0.401) |
| Guanajuato | 0.290 (0.015-0.062) | A1B | 0.042 (0.021-0.089) | 0.049 (0.024-0.101) | 0.060 (0.030-0.125) |
| Guanajuato | 0.290 (0.015-0.062) | A2 | 0.037 (0.019-0.080) | 0.047 (0.023-0.097) | 0.061 (0.031-0.125) |
| Guanajuato | 0.290 (0.015-0.062) | B1 | 0.040 (0.019-0.083) | 0.042 (0.021-0.087) | 0.052 (0.026-0.107) |
| Guerrero | 2.456 (1.745-3.610) | A1B | 2.779 (1.968-4.095) | 2.874 (2.031-4.264) | 2.982 (2.113-4.391) |
| Guerrero | 2.456 (1.745-3.610) | A2 | 2.616 (1.859-3.836) | 2.850 (2.016-4.218) | 2.950 (2.074-4.359) |
| Guerrero | 2.456 (1.745-3.610) | B1 | 2.605 (1.844-3.836) | 2.851 (2.033-4.148) | 2.872 (2.023-4.233) |
| Hidalgo | 0.173 (0.102-0.308) | A1B | 0.256 (0.152-0.453) | 0.324 (0.189-0.573) | 0.417 (0.244-0.747) |
| Hidalgo | 0.173 (0.102-0.308) | A2 | 0.228 (0.135-0.405) | 0.310 (0.181-0.546) | 0.439 (0.258-0.776) |
| Hidalgo | 0.173 (0.102-0.308) | B1 | 0.245 (0.144-0.441) | 0.278 (0.163-0.476) | 0.353 (0.210-0.617) |
| Jalisco | 0.634 (0.437-0.962) | A1B | 0.842 (0.584-1.266) | 0.977 (0.679-1.476) | 1.044 (0.728-1.576) |
| Jalisco | 0.634 (0.437-0.962) | A2 | 0.780 (0.540-1.172) | 0.918 (0.637-1.386) | 1.089 (0.759-1.624) |
| Jalisco | 0.634 (0.437-0.962) | B1 | 0.804 (0.552-1.219) | 0.849 (0.593-1.266) | 0.973 (0.668-1.460) |
| Mexico | 0.006 (0.002-0.016) | A1B | 0.011 (0.004-0.029) | 0.014 (0.005-0.039) | 0.020 (0.007-0.055) |
| Mexico | 0.006 (0.002-0.016) | A2 | 0.009 (0.003-0.025) | 0.013 (0.005-0.037) | 0.022 (0.009-0.061) |
| Mexico | 0.006 (0.002-0.016) | B1 | 0.010 (0.004-0.028) | 0.011 (0.004-0.029) | 0.015 (0.006-0.042) |
| Michoacan | 0.595 (0.397-0.918) | A1B | 0.754 (0.505-1.161) | 0.892 (0.601-1.375) | 0.988 (0.665-1.507) |
| Michoacan | 0.595 (0.397-0.918) | A2 | 0.707 (0.474-1.085) | 0.827 (0.554-1.280) | 0.970 (0.662-1.497) |
| Michoacan | 0.595 (0.397-0.918) | B1 | 0.722 (0.488-1.114) | 0.777 (0.524-1.189) | 0.898 (0.603-1.382) |
| Morelos | 1.576 (1.081-2.360) | A1B | 1.865 (1.285-2.799) | 2.035 (1.395-3.091) | 2.125 (1.453-3.264) |
| Morelos | 1.576 (1.081-2.360) | A2 | 1.742 (1.198-2.614) | 1.915 (1.312-2.905) | 2.145 (1.467-3.292) |
| Morelos | 1.576 (1.081-2.360) | B1 | 1.827 (1.245-2.793) | 1.895 (1.307-2.854) | 2.039 (1.393-3.077) |
| Nayarit | 3.187 (2.187-4.864) | A1B | 3.623 (2.479-5.568) | 3.647 (2.483-5.658) | 3.256 (2.239-4.993) |
| Nayarit | 3.187 (2.187-4.864) | A2 | 3.377 (2.323-5.153) | 3.247 (2.223-4.997) | 3.278 (2.235-5.045) |
| Nayarit | 3.187 (2.187-4.864) | B1 | 3.368 (2.313-5.165) | 3.451 (2.371-5.276) | 3.120 (2.138-4.690) |
| Nuevo Leon | 1.683 (1.141-2.589) | A1B | 2.092 (1.427-3.208) | 2.296 (1.555-3.510) | 2.539 (1.757-3.874) |
| Nuevo Leon | 1.683 (1.141-2.589) | A2 | 2.001 (1.360-3.082) | 2.240 (1.520-3.430) | 2.654 (1.801-4.043) |
| Nuevo Leon | 1.683 (1.141-2.589) | B1 | 1.950 (1.330-2.997) | 2.248 (1.539-3.399) | 2.392 (1.617-3.601) |
| Oaxaca | 1.737 (1.224-2.559) | A1B | 2.098 (1.478-3.098) | 2.378 (1.671-3.477) | 2.685 (1.897-3.950) |
| Oaxaca | 1.737 (1.224-2.559) | A2 | 2.038 (1.436-3.002) | 2.335 (1.636-3.427) | 2.746 (1.920-4.062) |
| Oaxaca | 1.737 (1.224-2.559) | B1 | 0.212 (1.481-3.093) | 2.161 (1.535-3.128) | 2.455 (1.726-3.593) |
| Puebla | 0.239 (0.158-0.375) | A1B | 0.341 (0.255-0.535) | 0.432 (0.287-0.675) | 0.548 (0.358-0.866) |
| Puebla | 0.239 (0.158-0.375) | A2 | 0.310 (0.204-0.486) | 0.407 (0.269-0.636) | 0.574 (0.380-0.899) |
| Puebla | 0.239 (0.158-0.375) | B1 | 0.341 (0.225-0.537) | 0.360 (0.238-0.555) | 0.463 (0.302-0.717) |
| Queretaro | 0.042 (0.014-0.138) | A1B | 0.056 (0.018-0.179) | 0.067 (0.022-0.227) | 0.082 (0.026-0.278) |
| Queretaro | 0.042 (0.014-0.138) | A2 | 0.053 (0.017-0.181) | 0.067 (0.020-0.223) | 0.085 (0.026-0.274) |
| Queretaro | 0.042 (0.014-0.138) | B1 | 0.056 (0.018-0.200) | 0.062 (0.194-0.202) | 0.072 (0.023-0.251) |
| Quintana Roo | 3.130 (2.19-4.674) | A1B | 3.383 (2.374-5.049) | 3.719 (2.584-5.522) | 3.742 (2.649-5.594) |
| Quintana Roo | 3.130 (2.19-4.674) | A2 | 3.207 (2.241-4.800) | 3.663 (2.555-5.424) | 4.055 (2.856-6.004) |
| Quintana Roo | 3.130 (2.19-4.674) | B1 | 3.390 (2.357-5.051) | 3.544 (2.497-5.262) | 3.632 (2.534-5.387) |
| San Luis Potosi | 0.845 (0.576-1.285) | A1B | 1.054 (0.720-1.598) | 1.188 (0.803-1.797) | 1.306 (0.884-1.997) |
| San Luis Potosi | 0.845 (0.576-1.285) | A2 | 1.006 (0.686-1.525) | 1.157 (0.783-1.748) | 1.374 (0.945-2.105) |
| San Luis Potosi | 0.845 (0.576-1.285) | B1 | 1.047 (0.713-1.613) | 1.091 (0.751-1.645) | 1.221 (0.833-1.845) |
| Sinaloa | 2.868 (2.025-4.240) | A1B | 3.095 (2.209-4.540) | 3.099 (2.202-4.561) | 3.022 (2.154-4.446) |
| Sinaloa | 2.868 (2.025-4.240) | A2 | 2.952 (2.109-4.306) | 2.910 (2.066-4.276) | 2.972 (2.112-4.393) |
| Sinaloa | 2.868 (2.025-4.240) | B1 | 2.871 (2.042-4.239) | 3.056 (2.190-4.473) | 3.046 (2.167-4.471) |
| Sonora | 1.211 (0.820-1.881) | A1B | 1.432 (0.984-2.153) | 1.518 (1.053-2.313) | 1.676 (1.181-2.519) |
| Sonora | 1.211 (0.820-1.881) | A2 | 1.332 (0.912-2.022) | 1.424 (0.982-2.186) | 1.747 (1.237-2.600) |
| Sonora | 1.211 (0.820-1.881) | B1 | 1.346 (0.922-2.087) | 1.425 (0.987-2.160) | 1.460 (1.003-2.204) |
| Tabasco | 3.370 (2.366-4.925) | A1B | 3.363 (2.378-4.887) | 4.592 (3.313-6.671) | 4.206 (3.020-6.191) |
| Tabasco | 3.370 (2.366-4.925) | A2 | 3.670 (2.579-5.383) | 3.923 (2.786-5.767) | 4.229 (3.030-6.156) |
| Tabasco | 3.370 (2.366-4.925) | B1 | 3.636 (2.534-5.433) | 3.854 (2.759-5.595) | 4.150 (2.951-6.047) |
| Tamaulipas | 3.039 (2.117-4.592) | A1B | 3.452 (2.416-5.191) | 3.782 (2.620-5.669) | 4.208 (2.840-5.999) |
| Tamaulipas | 3.039 (2.117-4.592) | A2 | 3.435 (2.408-5.160) | 3.671 (2.542-5.507) | 4.028 (2.810-5.984) |
| Tamaulipas | 3.039 (2.117-4.592) | B1 | 3.417 (2.374-5.179) | 3.615 (2.530-5.360) | 3.896 (2.714-5.802) |
| Tlaxcala | 0.069 (0.032-0.160) | A1B | 0.142 (0.065-0.322) | 0.203 (0.095-0.470) | 0.325 (0.146-0.735) |
| Tlaxcala | 0.069 (0.032-0.160) | A2 | 0.116 (0.053-0.264) | 0.192 (0.088-0.447) | 0.347 (0.162-0.777) |
| Tlaxcala | 0.069 (0.032-0.160) | B1 | 0.128 (0.061-0.293) | 0.148 (0.069-0.340) | 0.231 (0.107-0.529) |
| Veracruz | 2.630 (1.801-3.961) | A1B | 3.358 (2.292-5.067) | 3.388 (2.653-5.901) | 4.470 (3.104-6.836) |
| Veracruz | 2.630 (1.801-3.961) | A2 | 3.005 (2.067-4.568) | 3.377 (2.556-5.731) | 4.289 (2.934-6.578) |
| Veracruz | 2.630 (1.801-3.961) | B1 | 3.250 (2.235-4.892) | 3.522 (2.399-5.363) | 4.216 (2.823-6.345) |
| Yucatan | 2.185 (1.534-3.224) | A1B | 2.205 (1.544-3.243) | 2.296 (1.599-3.394) | 2.232 (1.573-3.290) |
| Yucatan | 2.185 (1.534-3.224) | A2 | 2.053 (1.434-3.008) | 2.274 (1.587-3.352) | 2.335 (1.651-3.461) |
| Yucatan | 2.185 (1.534-3.224) | B1 | 2.357 (1.651-3.491) | 2.321 (1.637-3.431) | 2.301 (1.613-3.409) |
| Zacatecas | 0.029 (0.010-0.092) | A1B | 0.041 (0.014-0.128) | 0.050 (0.016-0.154) | 0.062 (0.020-0.202) |
| Zacatecas | 0.029 (0.010-0.092) | A2 | 0.038 (0.013-0.117) | 0.048 (0.015-0.155) | 0.066 (0.022-0.211) |
| Zacatecas | 0.029 (0.010-0.092) | B1 | 0.038 (0.012-0.124) | 0.043 (0.014-0.137) | 0.052 (0.017-0.155) |
